# Supplementary material for: Differential responses of the gut microbiome and resistome to antibiotic exposures in infants and adults
Source: Nat Commun. 2023 Dec 22;14:8526. doi: 10.1038/s41467-023-44289-6 (PMC10746713; doi:10.1038/s41467-023-44289-6)
Supplement: Supplementary file 1 — Supplementary Information [file 41467_2023_44289_MOESM1_ESM.pdf]

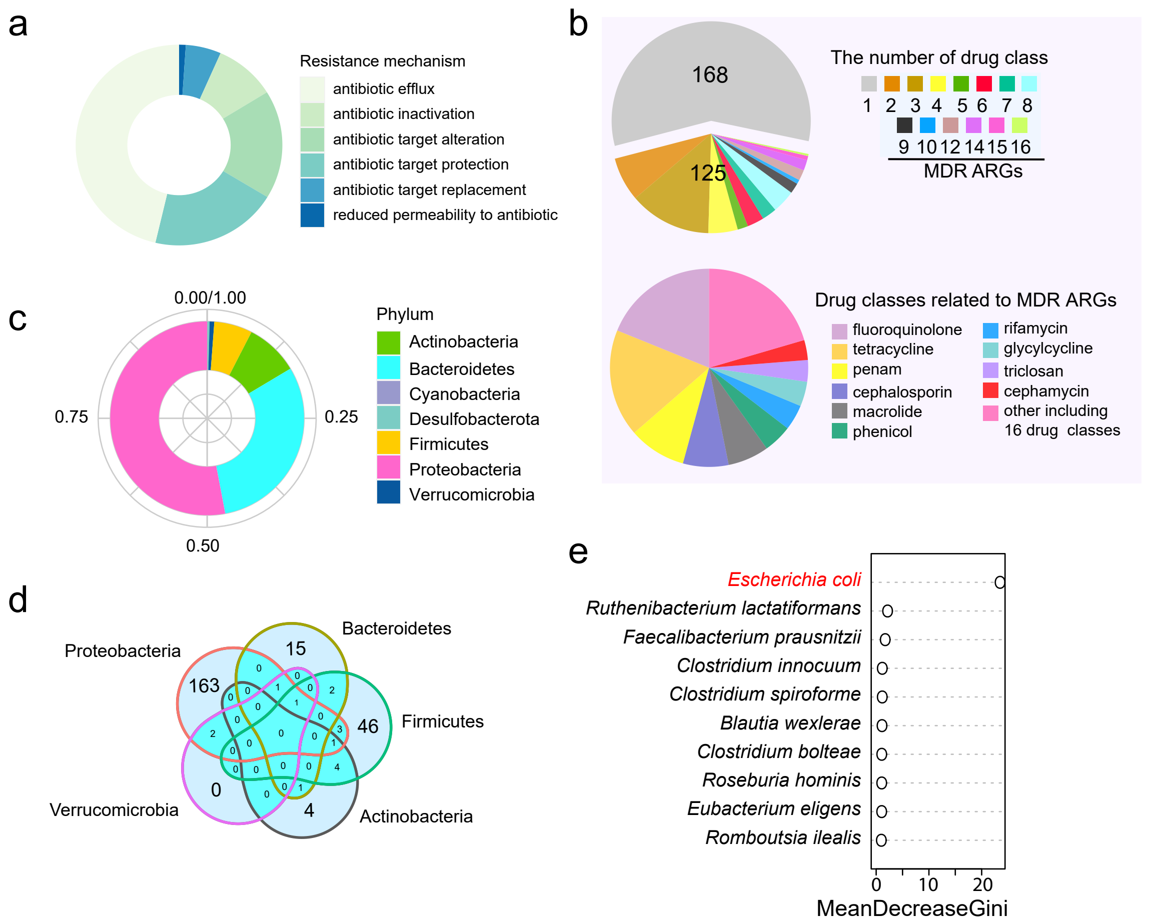

Fig. S1 ARG distribution in the adult gut and importance ranking of bacteria in shaping the two PAM clusters. a) Abundance proportion of ARGs with various resistance mechanisms. **b)** The number of MDR ARGs detected in the cohort (top panel) and abundance proportion of MDR ARGs with various resistant drug classes (bottom panel). **c)** Total abundance percent of ARGs in seven bacterial phyla in this cohort. **d)** Venn diagram demonstrating the numbers of overlapping and unique ARGs among five major bacterial phyla. **e)** Level of importance of bacterial species in shaping two PAM clusters by a Random Forest-based approach represented by the mean decrease in Gini. PAM clusters indicate the clustering of ARG composition based on a 'partitioning around medoids' (PAM) clustering analysis. The species are listed in order of importance from top to bottom.

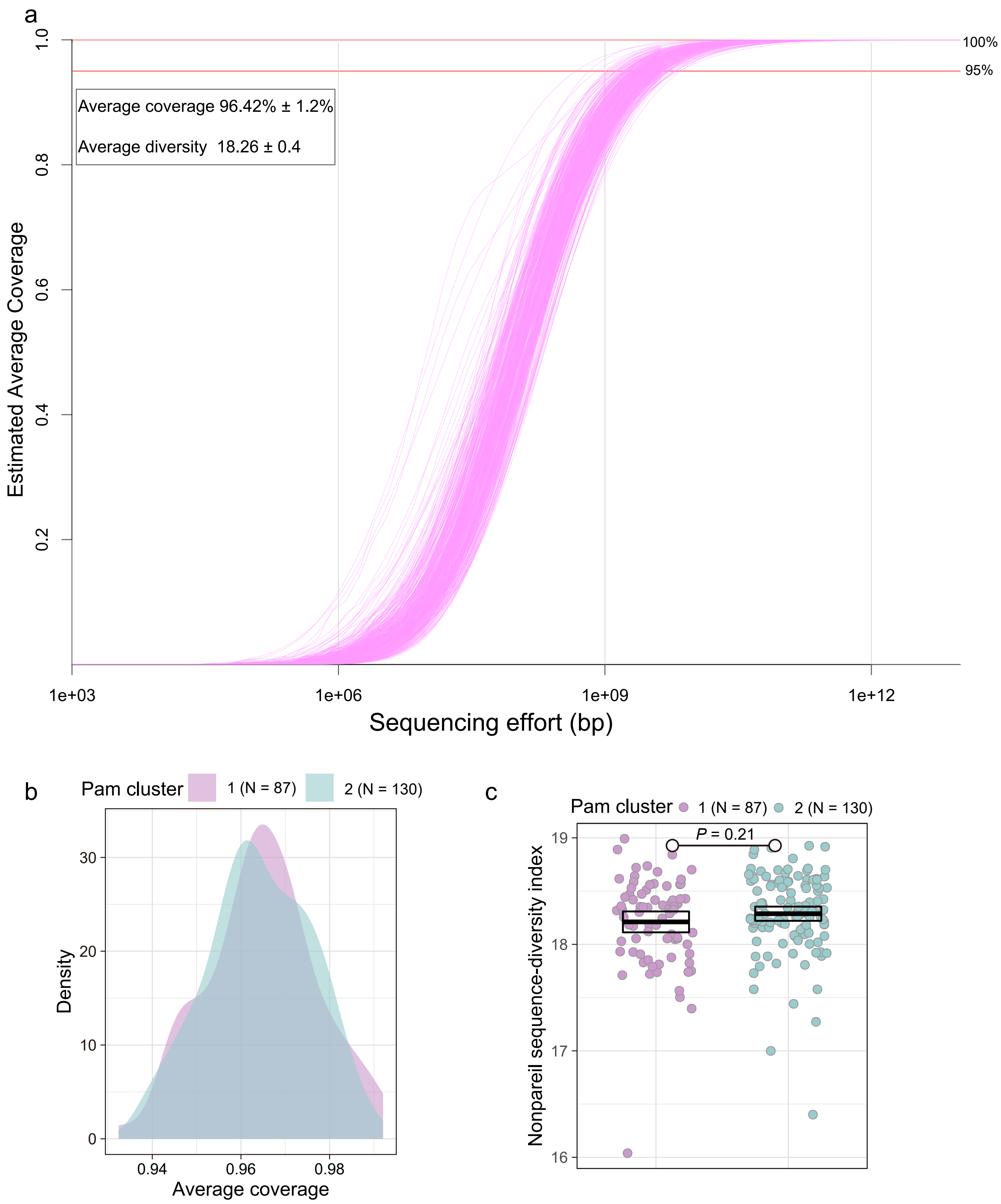

Fig. S2 Metagenomics sequencing coverage of adult samples assessed by Nonpareil using a k-mer kernel. a) Nonpareil curves showing the average coverage of all samples with sequencing effort. Average coverage and diversity (mean ± standard deviation) for all samples are shown. **b)** Density plot of average coverage in two PAM clusters. **c)** Sequence diversity in two PAM clusters. *P*-values correspond to the Wilcoxon test.

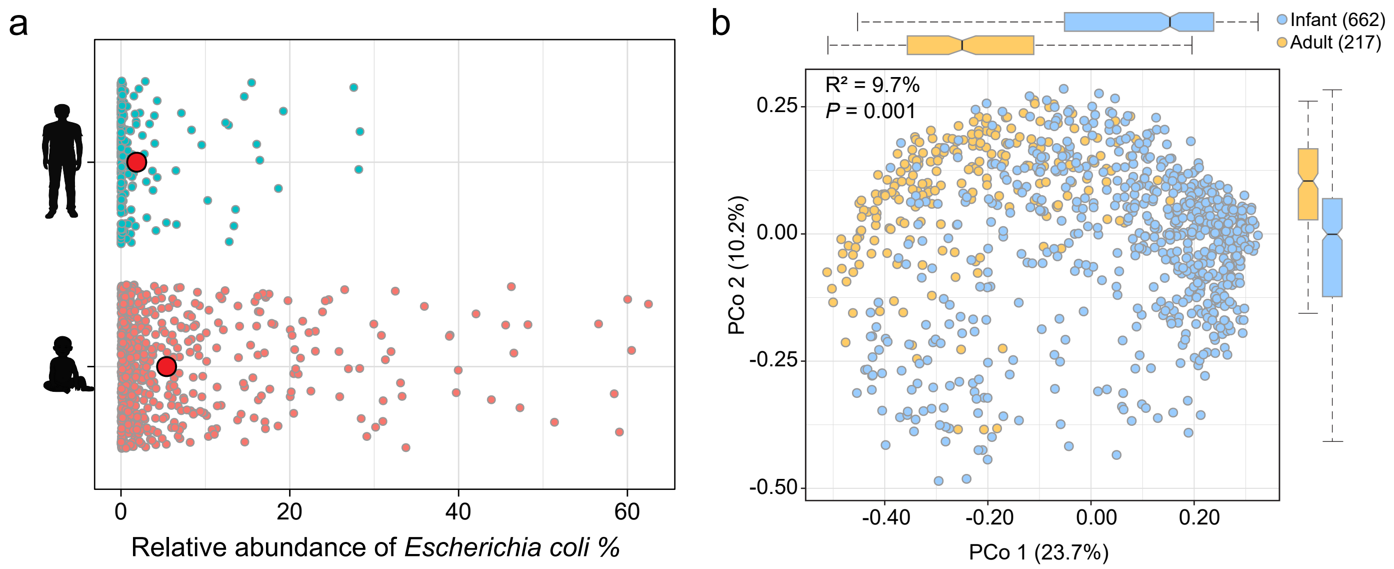

Fig. S3 The relative abundance of E. coli in adult and infant samples (a) and PCoA plot based on Bray-Curtis dissimilarity matrices of ARG abundances after removing E. coli ARGs in the adult and infant gut (b).

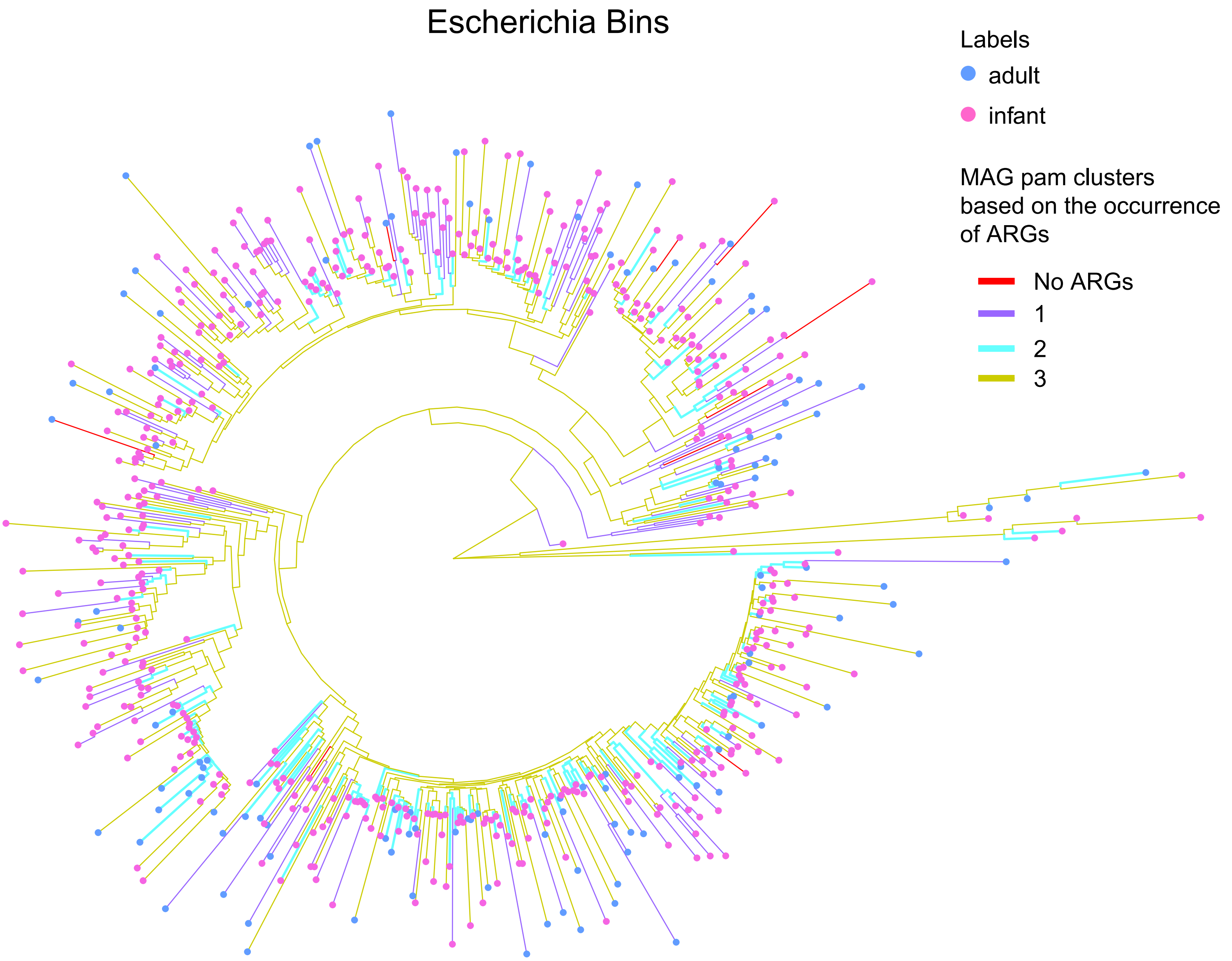

Fig. S4 Phylogenetic tree of Escherichia metagenome-assembled genomes (MAGs) in adult and infant gut based on 99% ANI analysis. *Escherichia* MAGs (with a length of over 200kb) demonstrated in the tree belong to a total of 7 species (*E. coli*, *E. coli*_D, *Shigella flexneri*, *E. dysenteriae*, *E. marmotae*, *E*. sp000208282, *E. albertii*). *Escherichia* MAGs are classified into four categories using PAM clustering based on the presence/absence of ARGs in MAGs. The different colored branches represent these three ARG profiles and MAGs without ARGs. MAGs from the four main sepcies including *E. coli*, *E. coli*_D, *Shigella flexneri*, *E. dysenteriae* were included in stastical analysis.

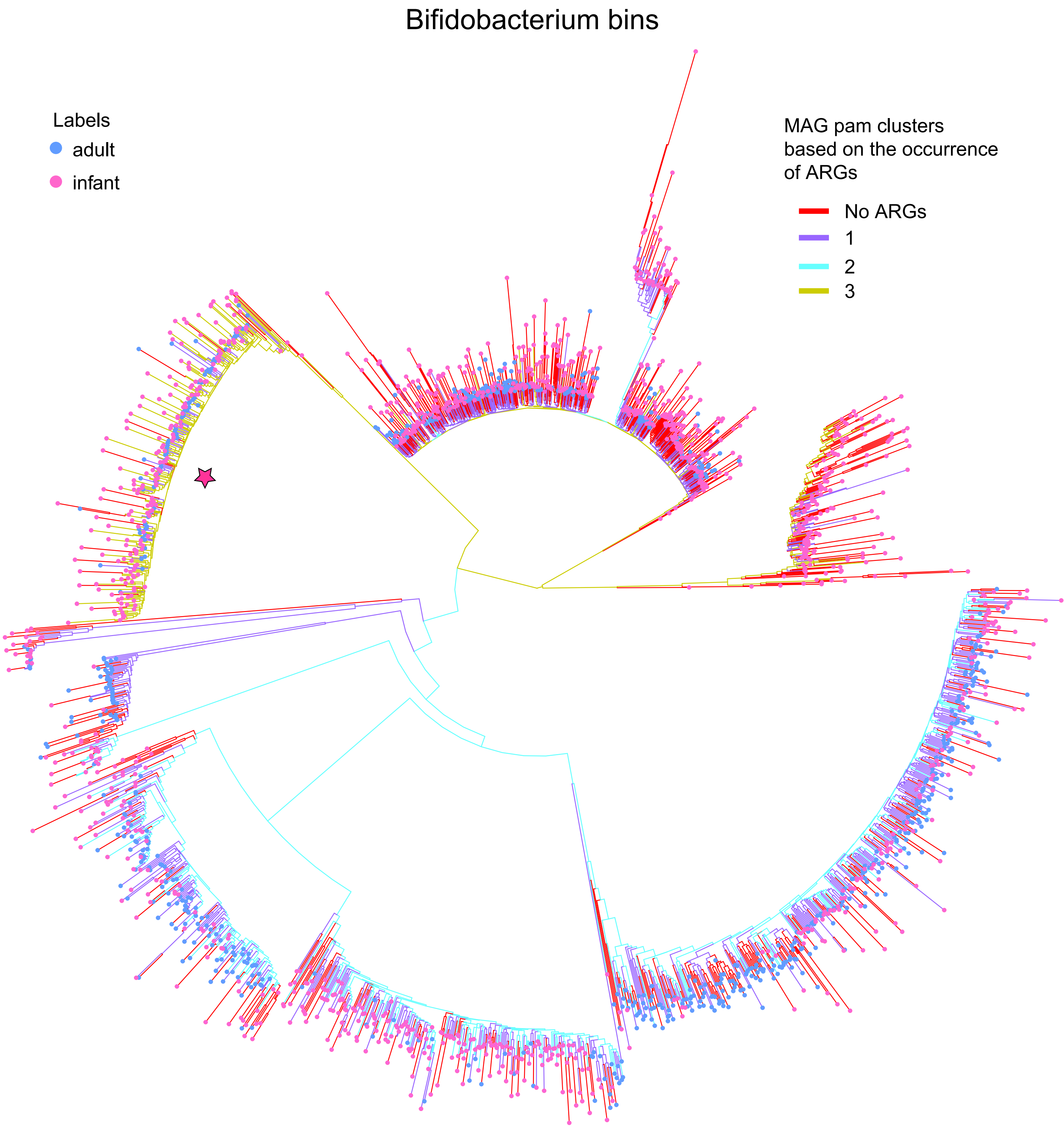

Fig. S5 Phylogenetic tree of Bifidobacterium metagenome-assembled genomes (MAGs) in adult and infant gut based on 99% ANI analysis. *Bifidobacterium* MAGs (with a length of over 200kb) demonstrated in the tree belong to a total of 8 species (*B. adolescentis*, *B. angulatum*, *B. bifidum*, *B. breve*, *B. catenulatum*, *B. infantis*, *B. longum*, *B. pseudocatenulatum*). *Bifidobacterium* MAGs are classified into four categories using PAM clustering based on the presence/absence of ARGs in MAGs. The different colored branches represent these three ARG profiles and MAGs without ARGs. ARG cluster 3 in infants is heavily distributed in one MAG cluster, marked with an asterisk.

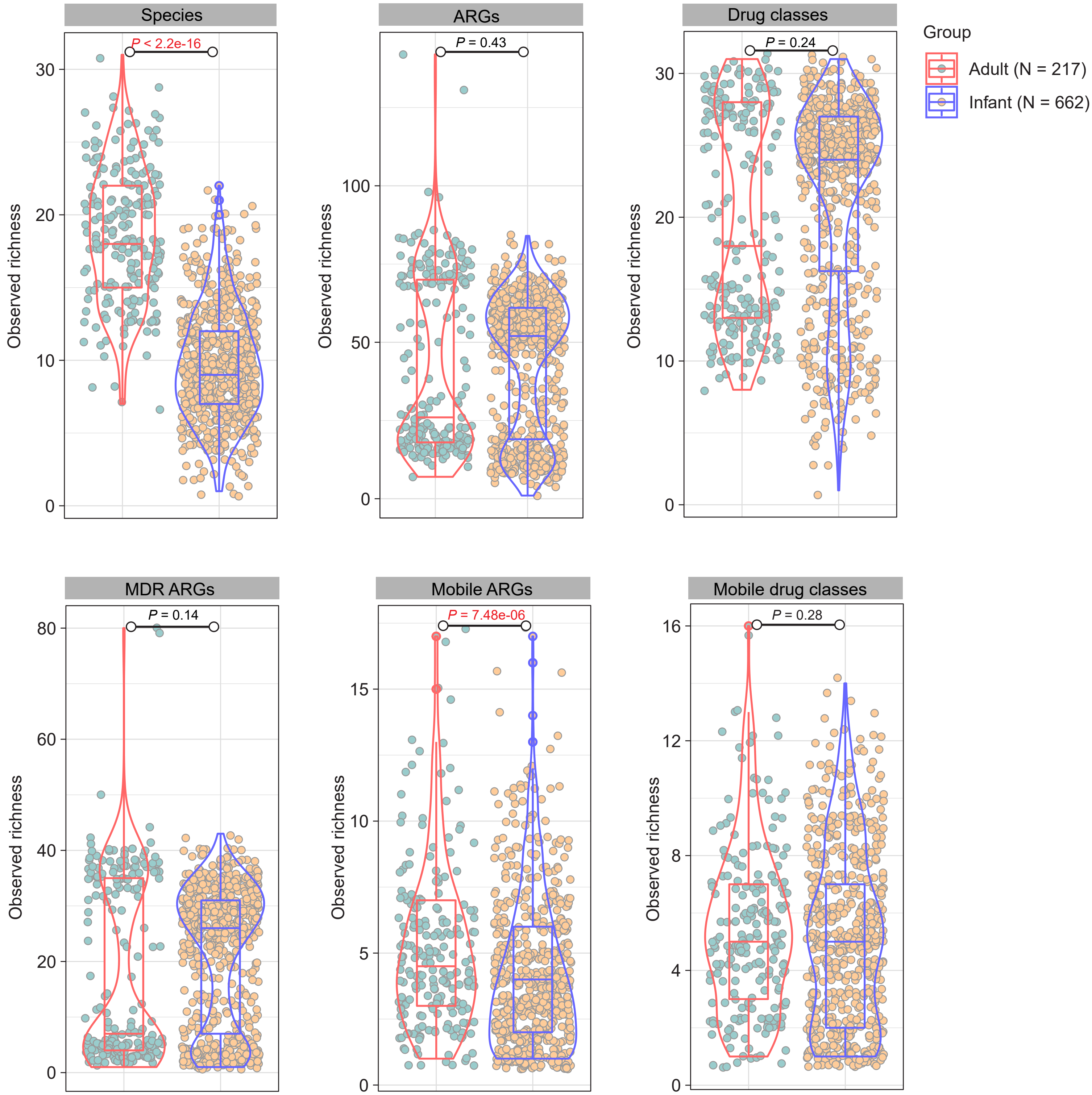

Fig. S6 The observed richness of bacterial species carrying ARGs, ARGs, drug classes, MDR ARGs, mobile ARGs, and mobile drug classes in the adult and infant gut, as a measure of alpha diversity. *P*-value obtained from the Wilcoxon test and red indicates *P* < 0.05 (significance difference).

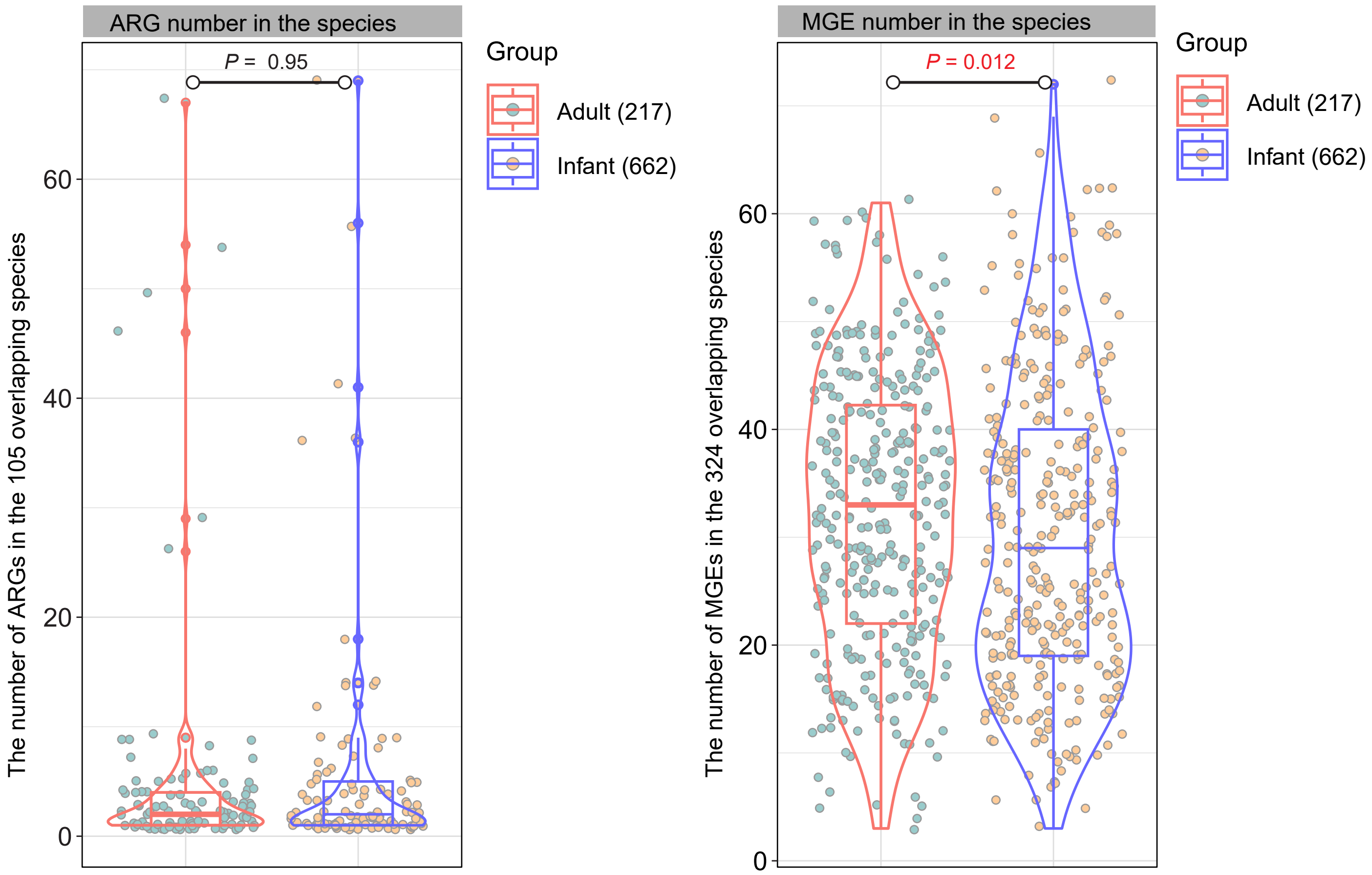

Fig. S7 The number of ARGs/MGEs in the shared ARG-carrying/MGE-carring bacterial species in the adult and infant gut. *P*-value obtained from the Wilcoxon test and red indicates $P < 0.05$ (significance difference).

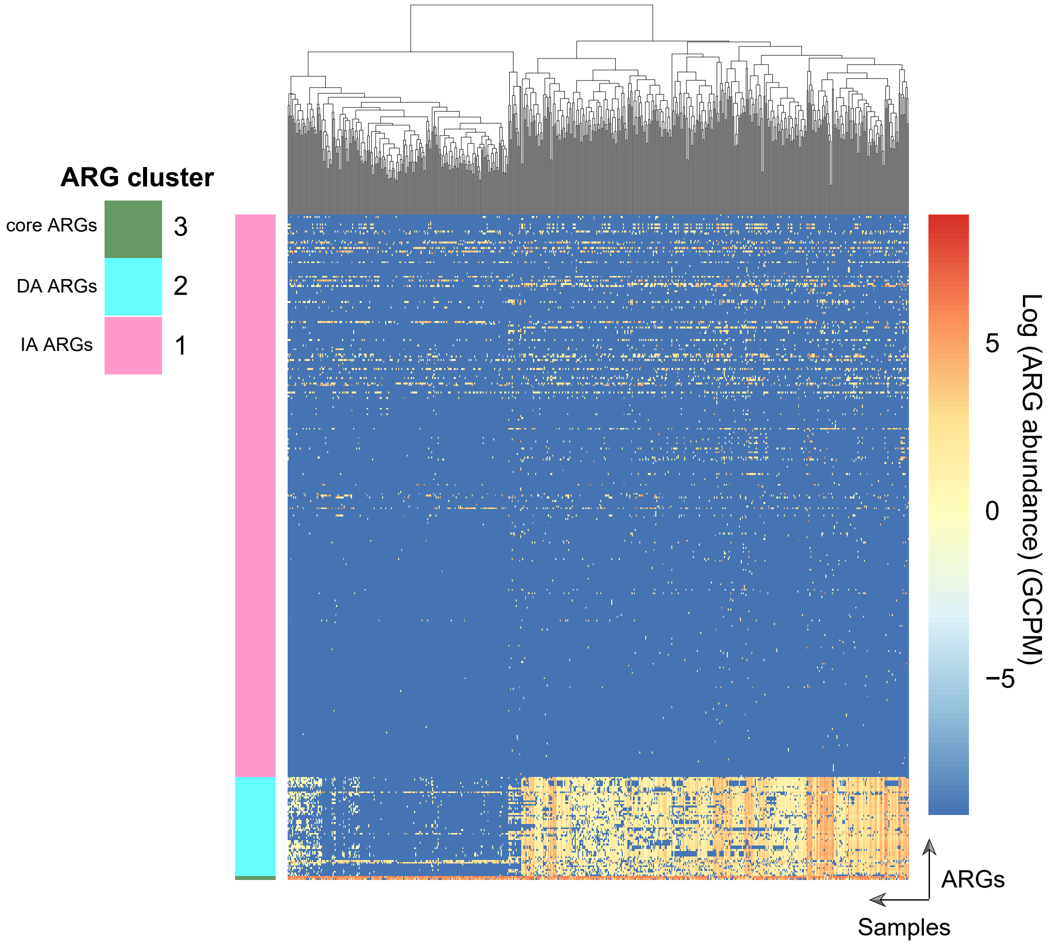

Fig. S8 Heatmap with the abundance of 366 ARGs across infant samples. Samples were clustered with Euclidean distance by complete linkage hierarchical clustering. ARGs were clustered into three categories with Euclidean distance by PAM clustering; Cluster 3 (core ARGs, N = 2) contains high abundant and prevalent ARGs in the samples. Cluster 2 (differentially abundance (DA) ARGs, N = 55) contains ARGs with significant abundance differences between samples. Cluster 1 (intermediate-abundance (IA) ARGs, N = 309) contains ARGs whose abundance in the samples falls between the ARGs in cluster 3 and those in cluster 2.

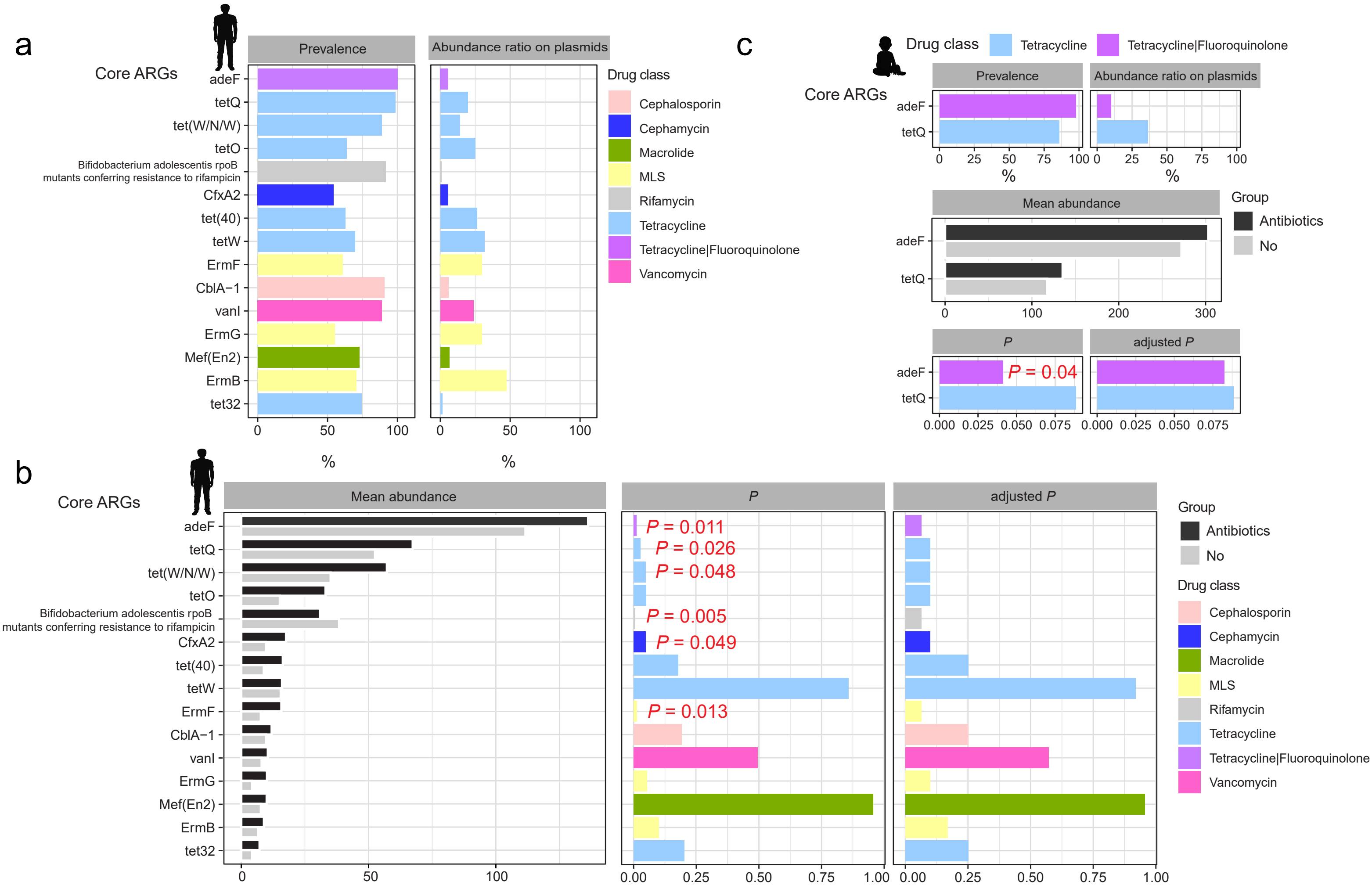

Fig. S9 An overview of core ARGs in the adult and infant gut and the impact of antibiotic treatment on core ARGs in both guts. a) Prevalence of core ARGs in the adult gut and the abundance proportion of core ARGs on plasmids. **b)** Effect of antibiotics on the mean abundance of core ARGs in the adult gut, and *P*-values and FDR-adjusted *P*-values obtained by the Wilcoxon test for comparisons. Red indicates $P < 0.05$ (significant difference) . **c)** Prevalence of core ARGs and the abundance proportion of core ARGs on plasmids in the infant gut, and the effect of antibiotics on the mean abundance of core ARGs and *P*-values and FDR-adjusted *P*-values obtained by the Wilcoxon test for comparisons. Red indicates $P < 0.05$ (significant difference).

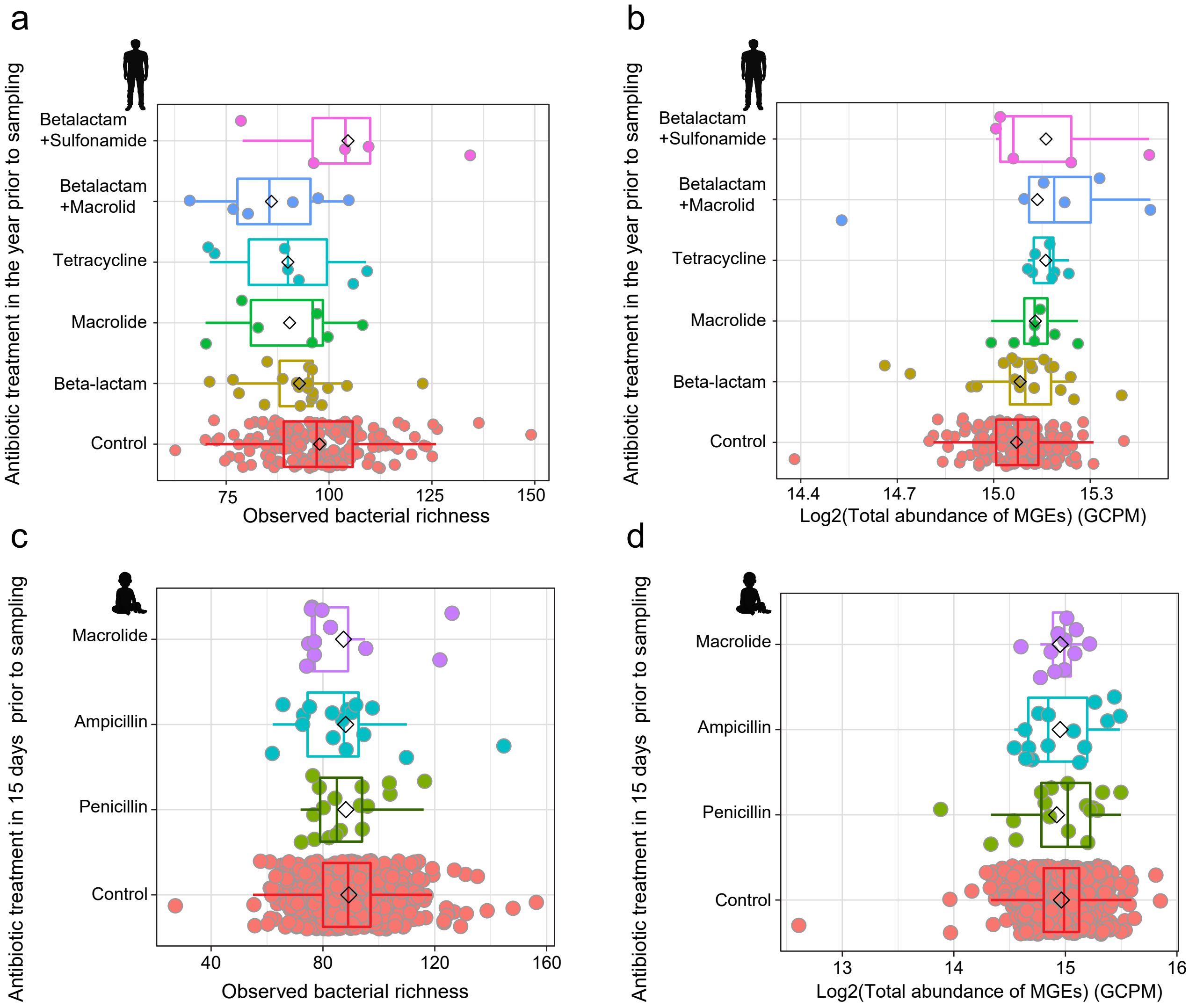

Fig. S10 The effects of various antibiotic exposures on bacterial observed richness and MGE abundance. a & b) Changes in bacterial observed richness **(a)** and MGE abundance **(b)** in the gut of adults who had taken five major antibiotics and antibiotic combinations in one year before sampling. Controls are those samples that had not taken antibiotics within one year. All *P*-values obtained by the Wilcoxon test adjusted with FDR are greater than 0.05, for all pairwise comparisons. **c & d)** Changes in bacterial observed richness **(c)** and MGE abundance **(d)** in the gut of infants who had taken three major antibiotics in 15 days before sampling. To exclude interactions between antibiotics, only samples that had taken a single antibiotic were included. Controls are those samples that had not taken antibiotics within 15 days before sampling. All *P*-values obtained by the Wilcoxon test adjusted with FDR are greater than 0.05, for all pairwise comparisons. The black diamond refers to the mean value.

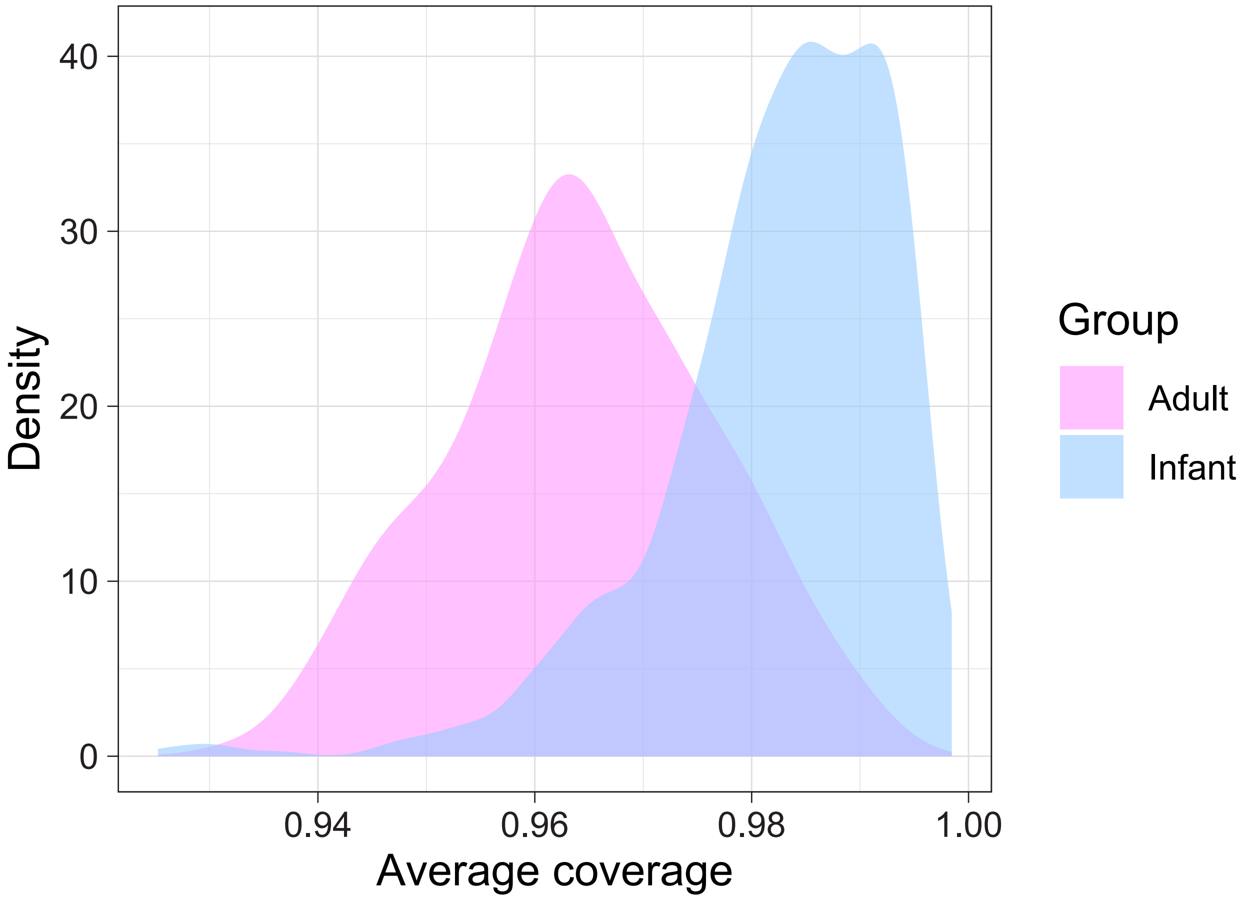

Fig. S11 Density plot of average coverage in adult and infant metagenomics samples calculated by Nonpareil using a k-mer kernel.
