# Supplementary material for: Differential responses of the gut microbiome and resistome to antibiotic exposures in infants and adults
Source: Nat Commun. 2023 Dec 22;14:8526. doi: 10.1038/s41467-023-44289-6 (PMC10746713; doi:10.1038/s41467-023-44289-6)
Supplement: Supplementary file 3 — Description of supplementary datasets [file 41467_2023_44289_MOESM3_ESM.pdf]

Description of Additional Supplementary Files

File Name: Supplementary Dataset 1

Description: The detailed distribution of various ARGs in bacteria species.

File Name: Supplementary Dataset 2

Description: The ARG patterns in 5 major bacterial phyla.

File Name: Supplementary Dataset 3

Description: The ARG clusters in adult and infant gut.

File Name: Supplementary Dataset 4

Description: The number of type of ARGs or MGEs in the shared ARG-carrying or MGE-carrying bacterial species.

File Name: Supplementary Dataset 5

Description: The shared and unique gut ARG characterization by adults and infants in six aspects.
