# Supplementary material for: Differential responses of the gut microbiome and resistome to antibiotic exposures in infants and adults
Source: Nat Commun. 2023 Dec 22;14:8526. doi: 10.1038/s41467-023-44289-6 (PMC10746713; doi:10.1038/s41467-023-44289-6)
Supplement: Supplementary file 9 — Reporting summary [file 41467_2023_44289_MOESM9_ESM.pdf]

nature research

Corresponding author(s): Søren J. Sørensen

Last updated by author(s): 2023-9-29

Reporting Summary

Nature Research wishes to improve the reproducibility of the work that we publish. This form provides structure for consistency and transparency in reporting. For further information on Nature Research policies, see Authors & Referees and the Editorial Policy Checklist .

Statistics

For all statistical analyses, confirm that the following items are present in the figure legend, table legend, main text, or Methods section.

n/a	Confirmed	
☐	☒	The exact sample size (*n*) for each experimental group/condition, given as a discrete number and unit of measurement
☐	☒	A statement on whether measurements were taken from distinct samples or whether the same sample was measured repeatedly
☐	☒	The statistical test(s) used AND whether they are one- or two-sided<br>*Only common tests should be described solely by name; describe more complex techniques in the Methods section.*
☐	☒	A description of all covariates tested
☐	☒	A description of any assumptions or corrections, such as tests of normality and adjustment for multiple comparisons
☐	☒	A full description of the statistical parameters including central tendency (e.g. means) or other basic estimates (e.g. regression coefficient) AND variation (e.g. standard deviation) or associated estimates of uncertainty (e.g. confidence intervals)
☐	☒	For null hypothesis testing, the test statistic (e.g. *F*, *t*, *r*) with confidence intervals, effect sizes, degrees of freedom and *P* value noted<br>*Give P values as exact values whenever suitable.*
☒	☐	For Bayesian analysis, information on the choice of priors and Markov chain Monte Carlo settings
☒	☐	For hierarchical and complex designs, identification of the appropriate level for tests and full reporting of outcomes
☐	☒	Estimates of effect sizes (e.g. Cohen's *d*, Pearson's *r*), indicating how they were calculated

Our web collection on statistics for biologists contains articles on many of the points above.

Software and code

Policy information about availability of computer code

Data collection	Data was collected during the visits to the clinical research unit or by the parents at home, following detailed instructions and stored in a dedicated custom online database. The data was double-checked against source data and subsequently locked. An audit trail was run routinely.
Data analysis	Metagenomics sequencing: Bioinformatic preprocessing was parallelized using GNU Parallel version 20180722. Sequencing adapters were removed using BBDuk, from BBTools version 38.19 (https://sourceforge.net/projects/bbmap/). Low-quality sequences and reads shorter than 50 bases were filtered out using Sickle version 1.33. Human contamination was filtered out using the BBMap feature of BBTools. Metagenomic diversity was analyzed using Nonpareil version 3.30, in kmer mode. Clean reads were assembled with SPAdes version 3.12.0. Contigs were binned into MAGs separately for each sample using Variational Autoencoders for Metagenomics Binning (VAMB). The taxonomic assignment of MAGs at least 200kbp was performed with the GTDB-tk v1.7.0 toolkit. Gene prediction was performed with prodigal (2.6.3) in META mode.<br><br>Antibiotic resistance gene and MGE predication and abundance calculation: Predicted genes were aligned using the Comprehensive Antibiotic Resistance Database (CARD version 3.0.7) through its accompanying resistance gene identifier (RGI) (https://card.mcmaster.ca/analyze/rgi). MGE homologs were characterized by HMM search in HMMER3 v3.1b2 in combination with the PFAM and TnpPred73 databases, with "cut_ga" as a threshold criterion. Clean reads were mapped against the predicted genes with Bowtie2 aligner. The number of mapped reads in bam files was calculated using Samtools idxstats of Samtools v1.12.<br><br>Plasmid prediction and calculation of contig abundance: Plasmid contigs were identified and characterized with Platon v5.3 using the default settings. Clean reads were mapped against the contigs with Bowtie2 aligner. The number of mapped reads in bam files was calculated using Samtools idxstats of Samtools v1.12.<br><br>Construction of phylogenetic tree of MAGs: The nucleotide-level similarity between MAGs assigned to Escherichia or Bifidobacterium was assessed with average nucleotide identity (ANI) values using FastANI v1.33. The neighbor-joining method was used to construct phylogenetic trees.

Statistics and data analysis: Data treatment and statistical analysis were conducted in the open-source statistical program “R” v4.1.2, including the R-package “ade4”, “artyfarty”, “alluvial”, “cluster”, ”dplyr”, “phyloseq”, "stats", “pheatmap", “extrafont”, “fpc”, “factoextra”, “grid”, “gridExtra”, “gtable”, “ggVennDiagram”, “ggplot2”, “ggrepel”, “ggalluvial”, “hrbrthemes”, “Nonpareil”, “reshape2”, “randomForest”, “rabuplot”, “stringr”, “scales”, “tidyr”, “tidyverse”, “vegan”, “viridis”.

For manuscripts utilizing custom algorithms or software that are central to the research but not yet described in published literature, software must be made available to editors/reviewers. We strongly encourage code deposition in a community repository (e.g. GitHub). See the Nature Research guidelines for submitting code & software for further information.

Data

Policy information about availability of data

All manuscripts must include a data availability statement. This statement should provide the following information, where applicable:

- Accession codes, unique identifiers, or web links for publicly available datasets
- A list of figures that have associated raw data
- A description of any restrictions on data availability

The COPSAC2010 metagenomics datasets are available in the Sequence Read Archive (SRA) under the accession number PRJNA715601. The COPSAC2000 metagenomics data have been deposited in the SRA under the accession number PRJNA916259.

Field-specific reporting

Please select the one below that is the best fit for your research. If you are not sure, read the appropriate sections before making your selection.

[x] Life sciences [ ] Behavioural & social sciences [ ] Ecological, evolutionary & environmental sciences

For a reference copy of the document with all sections, see nature.com/documents/nr-reporting-summary-flat.pdf

Life sciences study design

All studies must disclose on these points even when the disclosure is negative.

Sample size	The COPSAC2010 mother-child cohort forms the basis of an ongoing Danish cohort study of 736 pregnant women and 700 children who have been followed prospectively from week 24 of pregnancy. Fecal samples were collected from all children at age 1 week, 1 month, 1 year, 4 years, 5 years, 6 years and 10 year. The COPSAC2000 cohort is a mother-child cohort assembled for the primary purpose of studying asthma. The 217 fecal samples used for this study were collected as part of the 18-year follow-up visit at the research clinic or at home following detailed instructions.
Data exclusions	Samples with DNA concentration below 1 ng/ul in a volumn of 25 ul which is a minimum concentration for sequencing library preparation were excluded as their DNA were deemed unreliable and defined as failed.
Replication	We did not have access to a replication cohort.
Randomization	Not applicable since it is not relevant for the study.
Blinding	Not applicable.

Reporting for specific materials, systems and methods

We require information from authors about some types of materials, experimental systems and methods used in many studies. Here, indicate whether each material, system or method listed is relevant to your study. If you are not sure if a list item applies to your research, read the appropriate section before selecting a response.

Materials & experimental systems

n/a	Involved in the study	
[x]	[ ]	Antibodies
[x]	[ ]	Eukaryotic cell lines
[x]	[ ]	Palaeontology
[x]	[ ]	Animals and other organisms
[ ]	[x]	Human research participants
[x]	[ ]	Clinical data

Methods

n/a	Involved in the study	
[x]	[ ]	ChIP-seq
[x]	[ ]	Flow cytometry
[x]	[ ]	MRI-based neuroimaging

Human research participants

Policy information about studies involving human research participants

Population characteristics	The COPSAC2010 and COPSAC2000 mother-child cohort consists of participants (pregnant women and their children) recruited in Denmark on the island of Zealand. It is an unselected cohort thus reasonably reflecting mothers and children in the general Danish population.
Recruitment	Pregnant women were mailed and invited to participate in the cohort after the first pregnancy visit at their GP. For more details, see Bisgaard et al, Clin Exp Allergy 2013., doi 10.1111/cea.12213./Ann. Allergy, Asthma Immunol., 2004., doi:10.1016/S1081-889 1206(10)61398-1.
Ethics oversight	The study was designed with the guiding principles of the Declaration of Helsinki in mind and was approved by the Local Ethics Committee of the Danish Capital Region (COPSAC2000: KF 01-289/96, COPSAC2010: (H-B-2008-093)) and the Danish Data Protection Authority (both cohorts: 2015-41-3696).

Note that full information on the approval of the study protocol must also be provided in the manuscript.
